# Motivation of Teleworkers and Non-teleworkers in Times of COVID-19 in Spain: An Exploratory Study Using Non-parametric Analysis and Classification and Regression Trees

**DOI:** 10.3389/fpsyg.2022.852758

**Published:** 2022-06-10

**Authors:** Marina Romeo, Montserrat Yepes-Baldó, Laia Beltrà

**Affiliations:** Department of Social Psychology and Quantitative Psychology, Universitat de Barcelona, Barcelona, Spain

**Keywords:** teleworking, COVID-19 outbreak, employees’ motivation, classification and regression trees, non-parametric analysis

## Abstract

With the outbreak of COVID-19 in spring 2020, small, medium, and large companies were forced to cope with the unexpected circumstances. Faced by this health emergency, it was necessary to ensure that staff remained motivated and that they could continue to carry out their duties despite the obstacles. The main goal of this exploratory research was to characterize employees who teleworked and who did not, and their motivation during the lockdown. A total of 11,779 workers from different-sized companies in various sectors answered an *ad hoc* questionnaire. By using non-parametric comparisons and Classification and Regression Trees (CRTs), the results show differences in both the assessment of strategies put into practice by the companies and the level of motivation of teleworkers and non-teleworkers, with the latter being more highly motivated. Nonetheless, teleworkers assessed their companies’ strategies and the role of their managers and colleagues more positively. This research helps to understand how different sectors have dealt with the crisis, according to the degree of teleworking implemented in each sector, and to what extent the motivation of the employees has been affected. The analysis of the large amount of data obtained confirms the importance of the role of managers in sustaining the motivation of their subordinates in times of crisis. In this sense, it is necessary to develop managers’ competencies in order to develop and maintain relations of trust and support with their coworkers. On the other hand, it is necessary to foster employees’ sense of meaningfulness and responsibility at work in order to keep them motivated.

## Introduction

On 11 March 2020, the World Health Organization (WHO, 2020) declared COVID-19 a pandemic as a result of the morbidity and mortality rates. With regard to its severe economic repercussions, Jackson et al. (2020) point out:

The economic fallout from the pandemic raises the risks of a global economic recession with levels of unemployment not experienced since the Great Depression of the 1930s. The human costs in terms of lives lost will permanently affect global economic growth in addition to the cost of rising levels of poverty, lives upended, careers derailed, and increased social unrest. Global trade could also fall by 18%, depending on the depth and extent of the global economic downturn, exacting an especially heavy economic toll on trade-dependent developing and emerging economies (p.1).

At the European level, during this period, a set of measures aimed at safeguarding jobs and organizations was developed. It was based on the Pan-European Guarantee Fund established by the European Investment Bank, which set aside €200 billion to support companies (particularly SMEs). Furthermore, the member states have received help from the EU in order to implement short-time work schemes and protect jobs during the pandemic (Ahrendt et al., 2021).

One of the most widely used measures was the introduction of telework (working from home). Nevertheless, not all organizations and sectors dealt with this situation of uncertainty in the same way (KPMG, 2020). Thus, as indicated by the research carried out by the Bank of Spain (Hernández de Cos, 2020) and based on the data provided by the Spanish Ministry of Inclusion, Social Security and Migration, in spring 2020 the accommodation, food, and beverage, and leisure sectors that the ones affected by the greatest number of temporary redundancy schemes (*Expediente de Regulación Temporal de Empleo*—*ERTE*). One of the reasons could be the poor feasibility of teleworking. In contrast, the education, health, and commerce sectors showed significantly lower levels.

Although teleworking had gradually increased over the years anyway (Eurostat, 2018), the pandemic undoubtedly accelerated its implementation [International Labor Organization (ILO), 2020]. The state of emergency meant that the percentage of European employees who teleworked ranged from 15% to 40%. This percentage reached 60% in Finland, 50% in Luxembourg, Netherlands, Belgium, and Denmark, and over 40% in Ireland, Austria, Italy, and Sweden (Ahrendt et al., 2020). In Spain, although there were differences between the autonomous communities, the percentage of employees teleworking reached 14%. Although this was below the European average, it should be noted that this still amounted to a 74.2% increase compared to pre-COVID figures (Adecco Group Institute, 2021). On the other hand, as made clear by the Royal Decrees 8/2020 (Spanish Government, 2020a) and 15/2020 (Spanish Government, 2020b), this state of affairs could not be properly considered teleworking *per se*: it was a consequence of the COVID-19 containment measures and the time they that they remained in force.

Hatayama et al. (2020) and International Labor Organization (ILO, 2020) point out that, during this period, the two factors determining whether employees were able to work from home were, on the one hand, broadband Internet access, and on the other, whether they had a computer at home. Hence, the International Labor Organization (ILO, 2020) concludes that

in the countries where a large proportion of jobs are in sectors such as ICT, professional services, finance and insurance, and public administration sectors can mobilize a greater proportion of the workforce to work from home, whereas countries with a heavy reliance on sectors such as manufacturing, agriculture, construction, and tourism are less able to do so (p. 3).

In the next section, we present how the companies dealt with the pandemic and how were the working regulations in Spain. Secondly, we describe different studies on the impact of teleworking on work motivation during the lockdown. Results on these studies were not conclusive, therefore our main objective is to add more empirical evidence on this relationship.

Subsequently, the method and results are presented, organized on descriptive analysis, motivation classification trees (attending the global sample and teleworkers and non-teleworkers subsamples). Finally, we include the discussion and main conclusions derived from our research.

## Theoretical Background

Organizations tried to adapt to the new situation by taking measures that affected conditions of employment. Some of them included the suspension of contracts or the reduction of working hours. Other measures implied changes in management and job organization derived from the implementation of teleworking. Finally, companies introduced internal and external communication processes that serve to provide emotional support, and pass on work recommendations, prevention regulations, and business decisions (De Diego, 2020). However, research conducted with 470 full and part-time American employees revealed that only 43% believed that their organization had a plan to deal with a crisis of this nature (McQuivey, 2020) and that this uncertainty affected their motivation (Anwar et al., 2021).

In Spain, the Ministry of Work and Social Economy (2020) ruled that companies could deal with the pandemic by temporarily suspending their business activity. In some cases, when this was not possible, organizations were required to comply with Spanish Government (1995) on the prevention of occupational risks, and inform employees of the risks they were facing at work in relation to COVID-19, and the new working conditions aimed at coping with these risks. Specifically, the new working conditions revolved around the management and organization of new work arrangements (such as teleworking) and the health and prevention measures put in place. All these measures had the potential to affect working hours, the number of employees coworking in the same place at the same time, rules on hygiene, and so on. But according to the Spanish Ministry of Work and Social Economy (2020), they could have no effect on workers’ rights, such as salaries, compensation, or increased costs incurred by the employees’ need for new technological resources to do their jobs.

The results obtained for the impact on work motivation of the measures adopted by organizations during the lockdown are not conclusive. In the case of teleworking some authors affirm that it has resulted in an increase in employees’ motivation and improved performance (Gutiérrez Durán and Solano Araya, 2020). Other studies have analyzed how technology and the way employees manage it has an impact on their motivation. In this sense, Sapta et al. (2021) found that the adequacy of the technology had a positive effect on employees’ motivation. On the other hand, Panisoara et al. (2020) point out the importance of the employees’ perception of self-efficacy in relation to technological knowledge. It motivated them to continue working in the conditions imposed by the COVID-19 regulations. Nevertheless, other studies have obtained contrary results. For example, Hitka et al. (2021) conclude that the impact of COVID-19 negatively affected workers’ motivation in micro and small enterprises, and Sarfraz et al. (2021) affirm that these lower levels of motivation are derived from the sense of professional isolation associated with teleworking.

During this period, although it was impossible to continue working together physically, the new technologies made it possible to maintain communication between colleagues, middle and senior managers as regards preventive measures, policies, and work procedures. As indicated by Kim et al. (2021) when middle managers perceive negative coping strategies (such as impression management and *laissez-faire* leadership) among their superiors, this increases their sense of uncertainty, which in turn may lead to lower levels of motivation. However, the scientific literature has not analyzed the impact on employees of how their colleagues have coped with the pandemic. In our opinion, this is a vicarious learning process that makes it absolutely relevant (Bandura, 1977) and we consider that it is easier for employees to implement coping strategies that have proved effective among peers and colleagues.

The main goal of this exploratory research was to characterize employees who teleworked and who did not, and their motivation during the lockdown. Firstly, we analyzed and compared the profiles of these employees based on their sociodemographic variables (gender, age, and tenure), position (managers, middle managers, and employees), business sector, and size of the company. For this objective, the research questions were as follows:

*RQ1*: Are there differences between teleworkers and non-teleworkers regarding on sociodemographic variables (gender, age, and tenure), position (managers, middle managers, and employees), business sector, and size of the company?

*RQ2*: How did teleworkers assess their working conditions during the lockdown?

Secondly, we analyzed employees’ perceptions of the processes and procedures introduced during the first lockdown period by organizations, and the factors that helped to obtain the highest levels of motivation among teleworkers and non-teleworkers. Bearing in mind the nature of previous studies, the research questions in this study were as follows:

*RQ3*: How did employees value the set of processes and procedures developed by their employers during the lockdown, comparing those who worked from home and those who did not?

*RQ4*: How did employees assess the strategies used by colleagues, middle managers, and senior managers to cope with the pandemic, comparing those who worked from home and those who did not?

*RQ5*: Are there any differences between the factors that helped to motivate teleworkers and non-teleworkers during the lockdown?

The novelty of this research resides in two aspects: (a) it aimed to provide an understanding of how different sectors dealt with the crisis (b) it aimed to compare teleworkers—these being employees who had not previously worked from home but were obliged to do so by the state of emergency—with non-teleworkers.

## Materials and Methods

### Participants

A total sample of 11,779 employees, managers, and middle managers from 38 organizations, clients of a leading Spanish consulting firm, answered the questionnaire. Table 1 shows a description of the sample in terms of individual sociodemographic variables (gender, age, tenure, and position) and organizational variables (sector and enterprise size). Additionally, participants indicated whether they had previous experience in teleworking and whether they had to telework during the first months of the pandemic.

**Table 1:** Sociodemographic variables.

Indicator	Categories	*N*	%	Valid %
Actual teleworking	Yes	7,559	64.2%	64.2%
	No	4,220	35.8%	35.8%
Previous teleworking experience	Yes	1,551	13.2%	15.8%
	No	8,253	70.1%	84.2%
	Missing	1,975	16.8%	
Gender	Women	3,470	29.5%	58.55%
	Men	2,457	20.9%	41.45%
	Missing	5,852	49.7%	
Age	Under 25	158	1.3%	3.3%
	25–45	2,846	24.2%	59.3%
	46–55	1,357	11.5%	28.3%
	Over 55	437	3.7%	9.1%
	Missing	6,981	59.3%	
Tenure	<1 year	432	3.7%	13.6%
	1–5 years	1,217	10.3%	38.4%
	6–10 years	370	3.1%	11.7%
	More than 10 years	1,153	9.8%	36.3%
	Missing	8,607	73.1%	
Position	Managers	225	1.9%	2.1%
	Middle managers	2,404	20.4%	22.9%
	Employees	7,865	66.8%	74.9%
	Missing	1.285	10.9%	
Sector	Industry	3,026	25.7%	25.7%
	Distr. and consumption	2,320	19.7%	19.7%
	Services	3,538	30.0%	30.0%
	Education, public administration, and health	2,895	24.6%	24.6%
Size	Under 250	940	8%	8%
	250–500	2,743	23.3%	23.3%
	Over 500	8,096	68.7%	68.7%

As can be seen in Table 1, there are significant gaps, especially as regards variables, such as gender, age, and tenure, because some organizations refused to gather this data for confidentiality reasons.

Most of the participants worked in the service sector in organizations with more than 500 employees. They had mostly been working in their companies for between 5 and 10 years and 25% held a managerial position (managers or middle managers). The majority were women aged between 26 and 45 years old.

As regards teleworking, the majority did so during the lockdown but only 13.2% had previous experience of this work format.

### Procedure

In an extraordinary meeting held on 14 March 2020, the Spanish cabinet declared a state of emergency and decreed the lockdown, prioritizing remote work and suspending face-to-face educational activities. For that reason, the questionnaire was distributed online during the quarantine. The consulting firm contacted with the managers from the client’ companies to ask for their participation. Companies who accepted to participate send the questionnaire and the informed consent to their employees, and they answered by means of an external link (to assure confidentiality and anonymity).

### Instrument

An *ad hoc* questionnaire was developed with 16 items that analyzed the various aspects under study.

#### Organizational Measures Adopted

Following the instructions of the Ministry of Work and Social Economy (2020), Spanish organizations introduced a set of measures to cope with the pandemic: management of internal and external communication, work management, health and prevention measures, and working conditions and salaries. Participants assessed these measures on a five-point Likert scale, with 1 being “very negative” and 5 “very positive.” Cronbach’s alpha indicated good internal consistency of the scale (α = 0.87).

#### Coping

Employees’ perceptions of how senior and middle managers and their colleagues dealt with the situation generated by the lockdown were evaluated. This assessment included three Likert-type items, with 1 being “very negative” and 5 “very positive.” Cronbach’s alpha indicated good internal consistency of the scale (α = 0.87).

#### Teleworking Conditions and Results

Employees’ perceptions of the following conditions were evaluated: technological resources available when working from home, access to information, supervision of teleworking by managers, colleagues’ attitude and efficiency, and their own time management and overall satisfaction, and the results obtained (productivity, quality, and effectiveness). It consisted of seven Likert-type items, with 1 being “very negative” and 5 “very positive.” Only employees who had teleworked during the pandemic lockdown of spring 2020 answered these items. Cronbach’s alpha indicated good internal consistency of the scale (α = 0.84).

#### Motivation

Motivation was measured with a single-item “*Understanding by motivation the degree of energy, effort, and enthusiasm that a person is willing to put in into his/her work, what is your current motivation*?.” It used a five-point Likert scale ranging from 1 (very low) to 5 (very high).

Additionally, we collected indicators related to sociodemographic variables (gender, age, and tenure), position (managers, middle managers, and employees), business sector, and size of the company.

### Data Analysis

First, the distribution of teleworkers and non-teleworkers by sociodemographic variables was analyzed using the contingency coefficient. Secondly, descriptive statistics were calculated for the two groups and for the global sample. Non-parametric analyses were used to explore differences between these two groups of employees (*U* Mann–Whitney) on sociodemographic and organizational variables, (RQ1), employees’ perceptions of the set of processes and procedures developed by their employers during the lockdown (RQ3), and the strategies used by colleagues, middle managers, and senior managers to cope with the pandemic (RQ4). Thirdly, a comparison of the assessment of teleworking conditions according to sociodemographic and organizational variables was run (*U* Mann–Whitney; RQ2).

Finally, Classification and Regression Trees (CART) using SPSS 25 were performed to identify which variables, when considered simultaneously, better predicted the participants’ level of motivation (RQ5). We used CART as a statistical approach because it is preferable to other parametric approaches for identifying homogenous subgroups. Additionally, it has greater resistance to the effects of multicollinearity, outliers, and missing data, and it is useful for detecting higher-order interactions among predictors before determining which variables should be included in the model (Merkle and Shaffer, 2011).

We obtained the trees with the complete sample and subsequently split it into two groups—teleworkers and non-teleworkers. To avoid overestimation, the level of pruning was fixed at a standard deviation of one. Additionally, the minimum size for the parent node was fixed at 100 for the global sample and 50 for the subsamples, and at 50 and 10, respectively, for the child nodes.

In order to generate the decision tree that would allow the analysis of the predictive variables of motivation, this variable was re-categorized into two groups: employees who had scores below the median, amounting to a score of four out of five (*n* = 7,749, 66.3%), and those above it (*n* = 3,944, 33.7%). We used an Ntiles procedure in SPSS, which automatically split the sample into two groups based on percentile 50. Thus, participants with scores equal to or less than 4 were placed in the first group and those with scores of five in the second group.

The predictive variables included in the analyses were as follows: participants’ perception of organizational measures adopted, coping, teleworking conditions and results (only in the global tree and in the teleworkers’ tree), gender, age, tenure, position, sector, and company size.

## Results

### Descriptive Statistics

Firstly, we analyzed differences in terms of the proportion of teleworking employees according to sociodemographic and organizational variables (Table 2). The companies with the highest percentage of teleworkers were those with fewer than 250 employees and operating in the education, public administration, and health sectors.

**Table 2:** Percentage of teleworking employees by sociodemographic and organizational variables.

Indicator	Categories	% of teleworkers	Contingency coefficient
Enterprise size	Under 250 250–500 Over 500	86.7% 73.1% 58.5%	0.184^**^
Sector	Industry Distr. and consumption Services Education, PA, and health	71.6% 35.2% 61.9% 82.4%	0.32^**^
Position	Managers Middle managers Employees	93.8% 58.9% 63.5%	0.102^**^
Age	Under 25 25–45 46–55 Over 55	42.4% 66.4% 72.4% 70.7%	0.115^**^
Tenure	<1 year 1–5 years 6–10 years More than 10 years	43.5% 42.3% 43.2% 50.7%	0.077^**^
Gender	Women Men	67.4% 72.3%	0.052^**^

** *p <* *0.001*.

In relation to individual variables, the highest percentages of teleworkers were found among managers, employees over 46 years old, employees with more than 10-year experience in the same company, and men.

Table 3 shows the statistics related to the global sample, the teleworker and non-teleworker subsamples, and the statistical contrasts between these groups. In general terms, teleworkers had a better perception of the organizational measures taken to deal with the pandemic situation with the exception of the strategies that focused on communication with clients, which non-teleworkers rated more positively.

**Table 3:** Descriptive statistics and comparisons between teleworkers and non-teleworkers.

Indicators	Global sample			Teleworkers		Non-teleworkers		Contrast
	*N*	Mean	SD	Mean	SD	Mean	SD	
**Organizational measures adopted**								
Internal communication about the situation	11,454	3.77	0.984	3.8	0.95	3.73	1.04	0.01
Management and work organization measures	11,690	3.71	1.040	3.77	1.01	3.60	1.08	<0.001
Health and prevention measures	11,650	3.74	1.060	3.84	1.00	3.57	1.14	<0.001
Communication with clients	10,442	3.79	0.901	3.77	0.90	3.83	0.90	0.004
Labor and salary measures	10,827	3.75	1.118	3.78	1.11	3.68	1.13	<0.001
**Coping**								
Top managers	11,669	3.72	1.077	3.76	1.05	3.66	1.12	<0.001
Middle managers	11,650	3.94	1.016	3.98	0.99	3.87	1.06	<0.001
Employees	11,656	4.31	0.780	4.35	0.75	4.25	0.834	<0.001
Motivation	11,693	4	0.953	3.99	0.932	4.00	0.99	<0.001

Teleworkers also gave higher ratings than non-teleworkers when evaluating the coping strategies of their top managers, middle managers, and colleagues. However, motivation levels were slightly—but significantly—higher among non-teleworkers.

As regards teleworking conditions (Table 4), all scores were above 3.75, indicating a good perception in all cases. The lowest score was given to personal time management and the highest to the attitude/efficiency of coworkers while teleworking. Overall satisfaction with the situation was near to 4, indicating medium–high levels.

**Table 4:** Descriptive statistics for teleworking conditions (only teleworkers subsample).

Indicators	Teleworkers		
	*N*	Mean	SD
Technological means available to work from home	7,469	3.80	1.047
Access to information	7,474	4.07	0.912
The way supervisors coped with the situation	7,470	3.96	0.984
The attitude/efficiency of peers	7,277	4.31	0.766
Individual time management	7,521	3.75	1.042
Work productivity, quality, and effectiveness	7,513	4.04	0.869
Overall satisfaction with teleworking	7,477	3.98	0.944

Finally, a comparison of the assessment of teleworking conditions by sociodemographic and organizational variables were run (Table 5).

**Table 5:** Comparisons of the scores on teleworking by sociodemographic variables (teleworkers subsample).

		Technological means		Access to information		Supervisors’ role		Attitude of peers		Time management		Productivity, quality, and effectiveness		Overall satisfaction	
		Mean	Contrast	Mean	Contrast	Mean	Contrast	Mean	Contrast	Mean	Contrast	Mean	Contrast	Mean	Contrast
Ent. size	Under 250 250–500 Over 500	3.86 3.78 3.81	2.555 *p* = 0.279	4.18 4.03 4.08	14.061 *p* = 0.001	4.02 3.87 4.00	20.612 *p* < 0.001	4.31 4.33 4.31	2.596 *p* = 0.273	3.69 3.80 3.74	9.071 *p* = 0.011	4.09 4.07 4.02	7.511 *p* = 0.023	4.02 4.01 3.97	4.502 *p* = 0.105
Sector	Industry Distr. and consumption Services Education, PA, and health	3.98 3.95 3.86 3.55	223.082 *p* < 0.001	4.16 4.16 4.09 3.95	81.169 *p* < 0.001	4.01 4.02 3.99 3.89	27.939 *p* < 0.001	4.28 4.33 4.37 4.29	18.815 *p* < 0.001	3.94 3.87 3.94 3.36	441.856 *p* < 0.001	4.12 4.08 4.13 3.84	179.604 *p* < 0.001	4.10 4.00 4.13 3.75	228.608 *p* < 0.001
Position	Managers Middle managers Employees	4.19 3.91 3.73	64.532 *p* < 0.001	4.40 4.12 4.01	47.009 *p* < 0.001	4.32 4.04 3.92	39.342 *p* < 0.001	4.32 4.27 4.32	8.232 *p* = 0.016	3.66 3.71 3.74	1.816 *p* = 0.403	4.06 4.03 4.02	0.156 *p* = 0.925	4.03 4.02 3.97	1.929 *p* = 0.381
Age	Under 25 25–45 46–55 Over 55	3.78 3.75 3.78 3.74	0.584 *p* = 0.900	4.15 4.14 4.05 3.95	24.027 *p* < 0.001	4.09 3.98 3.97 3.97	3.5 *p* = 0.321	4.44 4.33 4.32 4.30	6.711 *p* = 0.082	3.99 3.61 3.66 3.80	13.013 *p* = 0.005	4.00 3.99 4.01 4.00	0.471 *p* = 0.925	3.90 3.91 3.95 3.97	0.233 *p* = 0.972
Tenure	<1 year 1–5 years 6–10 years More than 10 years	4.07 3.93 4.06 3.73	24.796 *p* < 0.001	4.40 4.20 4.22 4.02	36.108 *p* < 0.001	4.31 4.07 4.03 3.92	33.823 *p* < 0.001	4.49 4.34 4.40 4.24	22.462 *p* < 0.001	3.98 3.83 3.89 3.73	12.865 *p* = 0.005	4.21 4.15 4.20 3.99	22.279 *p* < 0.001	4.14 4.05 4.19 3.99	10.613 *p* = 0.014
Gender	Women Men	3.77 3.79	2015204.5 *p* = 0.488	4.10 4.05	1989487.0 *p* = 0.093	4.00 3.95	1985749.5 *p* = 0.095	4.35 4.30	1749876.0 *p* = 0.016	3.67 3.71	2022061.0 *p* = 0.325	4.02 3.99	2009720.5 *p* = 0.172	3.96 3.97	2032041.5 *p* = 0.495

#### Size

In general terms, differences were only observed in access to information for teleworking, the role of supervisors, employees’ time management, and aspects related to work productivity, quality, and effectiveness. Specifically, employees of companies with fewer than 250 employees were the ones who most highly rated access to information. In the case of companies with 250–500 employees, they perceived the role played by their supervisors more negatively than the rest, but they considered they managed their time better. Finally, in those companies with more than 500 employees, work productivity, quality, and effectiveness were valued more negatively. There were no significant differences in the global assessment of the teleworking experience.

#### Sector

The most positive evaluations of teleworking came from employees in the industrial, distribution and consumption, and service sectors, with small significant differences between them. Conversely, the worst evaluations were those of education, public administration, and health employees. A discrepancy in this trend was only observed in the case of the assessment of the attitude of colleagues, where industry and the education, administration, and health sectors once again obtained the worst scores. Regarding general satisfaction with teleworking, employees in the service and industrial sectors were the most satisfied (without significant differences between them), followed by those working in distribution and consumption, education, and administration, and with health workers in last place.

#### Position

Significant differences were observed in technological resources, access to information for teleworking, and the role of supervisors, where the lower the hierarchical scale the poorer the evaluation. In the case of the attitude of colleagues, middle managers gave this variable a significantly more negative rating than employees.

#### Age

The age groups did not differ in their assessment of technological resources, supervisors’ role, work productivity, quality and effectiveness, and their general satisfaction with the teleworking experience. Regarding access to information, those under 45 had a more positive perception than other employees. On the other hand, when it comes to managing personal time, the two outermost age groups (under 25 s and over 55 s) gave more positive evaluations than the rest of the employees.

#### Tenure

People with the longest tenure (more than 10 years) were the ones who least valued all aspects related to teleworking and indicated the lowest levels of satisfaction with teleworking. Few significant differences were observed between the rest of the groups, although those who had been in a company for <1 year rated access to teleworking information higher than those who had been in a company for between 1 and 5 years, and valued the role of the supervisor better than other employees.

#### Gender

Significant differences were only observed in relation to the attitude of their colleagues, with women being the ones who valued this variable more favorably.

### Motivation Classification Trees

#### Global Sample

**Figure 1:**
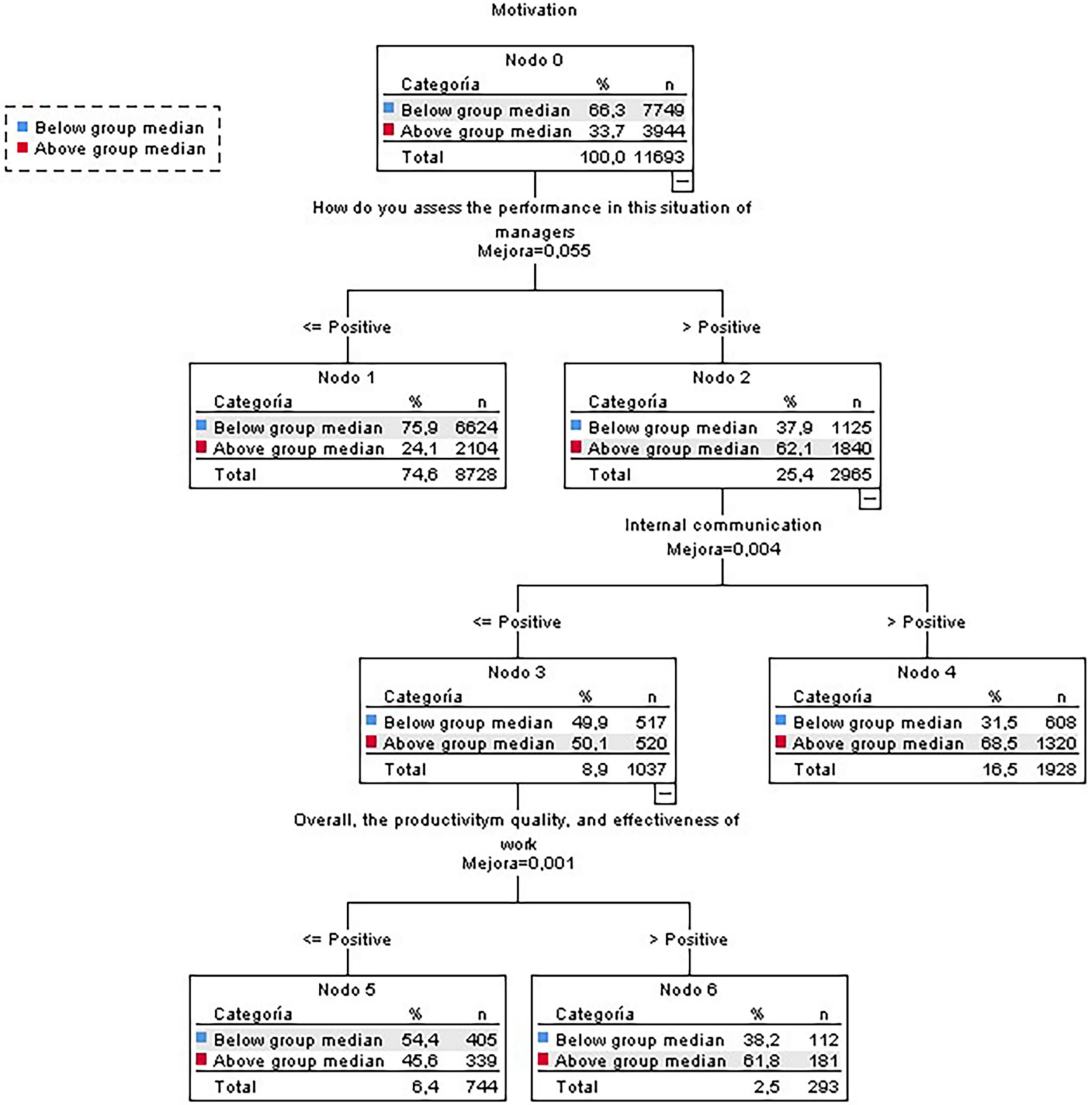
Classification tree for motivation (global sample).

The results indicate that the predictive variables of motivation were managers’ performance when coping with the situation (coherence, credibility, etc.), the perception of the internal communication of the situation, and the results obtained from teleworking in terms of productivity, quality, and effectiveness (Figure 1).

Terminal node 1 included a higher percentage of people with motivation scores below the median than those in node 0, amounting to 75.9% of the total node. This group included people who valued the performance of managers with scores equal to or less than 4 out of 5.

At the other extreme were terminal nodes 4 and 6, in which the percentages of people with scores above the median amounted to 68.5% and 61.8%, well above the initial 33.7%. Terminal node 4 included people who highly valued managers’ performance and the management of internal communication of the situation (with scores of 5). For its part, terminal node 6 included people who highly valued managers’ performance, although they gave scores of 4 or less to internal communication management. In addition, these participants also valued very positively (with scores of 5) the results obtained during teleworking in terms of productivity, quality, and effectiveness (this branch only includes employees who were teleworking).

The tree obtained had a global correct classification percentage of 72.9%, although it was found more suitable to classify participants with scores below the median (90.7%) rather than those above it (38.1%). It should be noted that none of the sociodemographic variables had enough discriminative power to appear in the classification tree.

#### Teleworker’s Subsample

**Figure 2:**
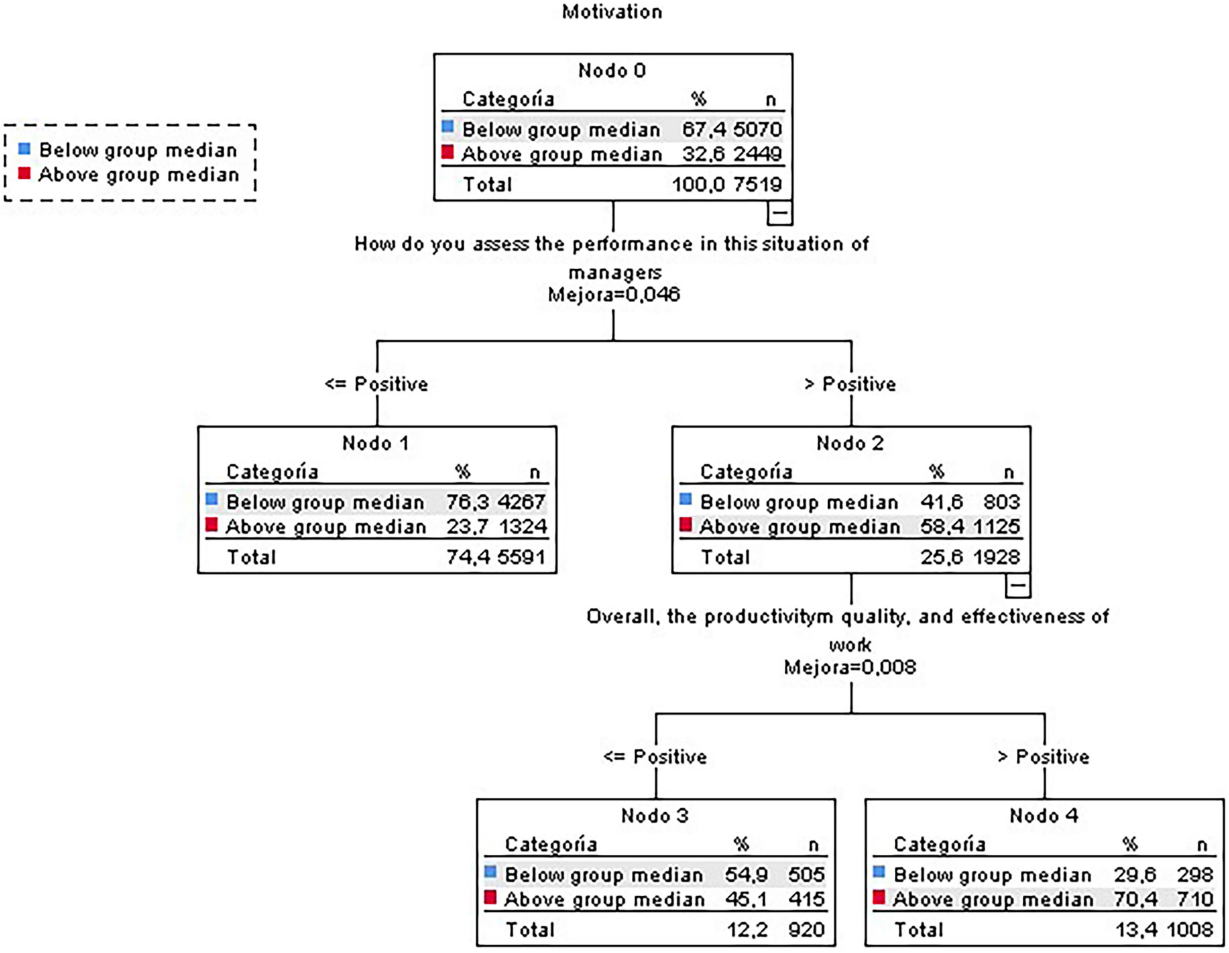
Classification tree for motivation (teleworkers subsample).

The tree for the teleworkers subsample was similar to the one for the general sample, but the variable “internal communication” was split out (Figure 2). The most highly motivated teleworkers were those who considered that their managers performed well when coping with the situation and were satisfied with the results they achieved. On the other hand, those employees below the group median on motivation were those who considered their managers’ performance average or worse.

The tree obtained had an overall correct classification percentage of 72.9% (participants classified in their motivational group—below/above median—correctly by the classification model), although it was found more suitable to classify participants with scores below the median (94.1%) rather than those above it (29%).

#### Non-teleworkers’ Subsample

**Figure 3:**
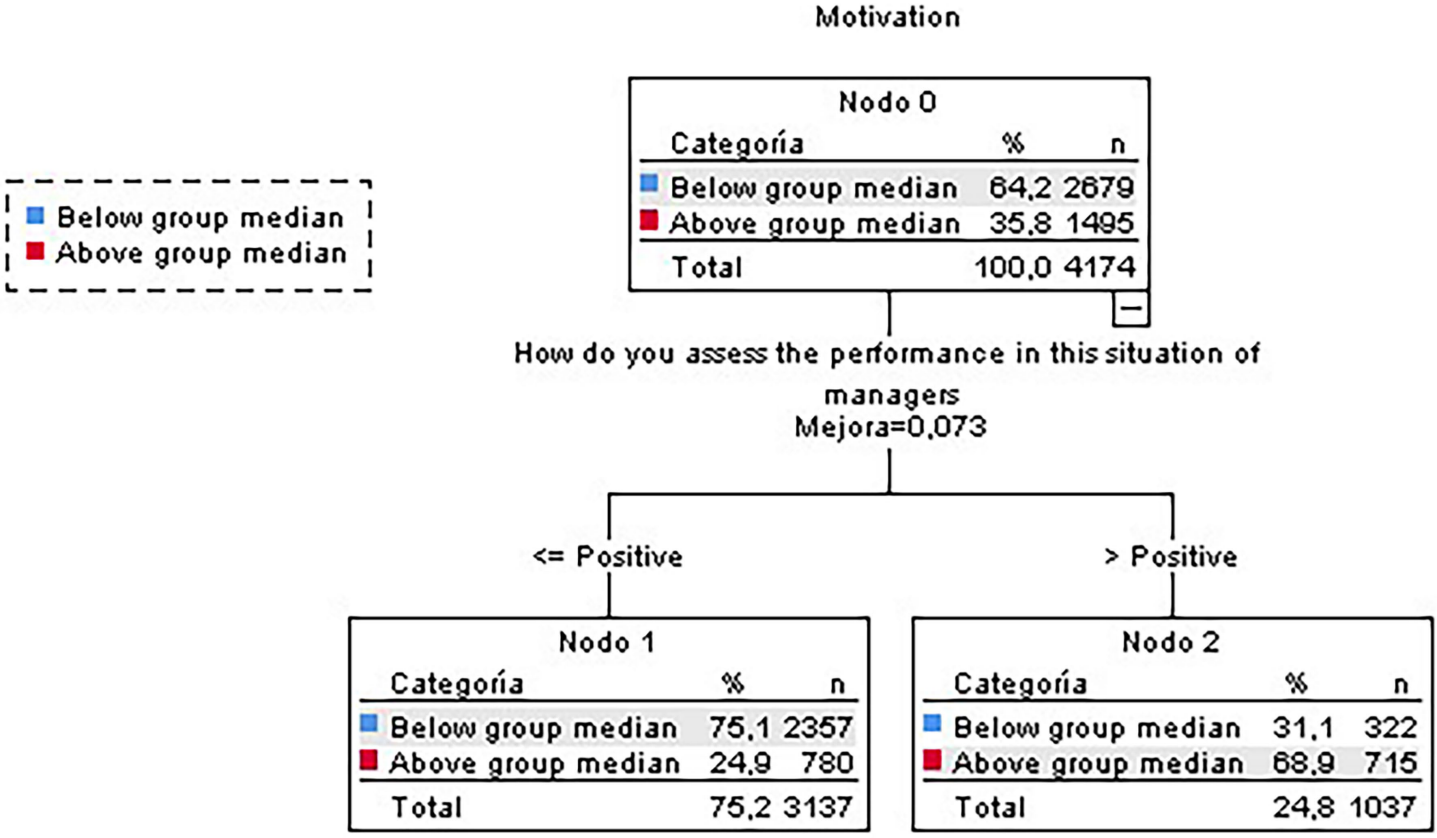
Classification tree for motivation (non-teleworkers subsample).

To obtain the classification tree for the non-teleworkers’ subsamples, all items related to teleworking conditions were excluded. The tree for the non-teleworkers’ subsample was the simplest obtained (Figure 3). In this case, the most highly motivated non-teleworkers were those who considered that their managers performed well when coping with the situation. Contrariwise, those employees below the group median for motivation were those who considered their managers’ performance average or worse.

The tree obtained had an overall correct classification percentage of 73.6%, although it was found more suitable to classify participants with scores below the median (88%) rather than those above it (47.8%).

## Discussion and Conclusions

The main goal of this exploratory research was to characterize employees who teleworked and who did not, and their motivation during the lockdown. A comparative analysis was carried out of data from more than 11,000 participants, working in 38 Spanish different-sized companies from various sectors. The sample provided a good approximation of the state of affairs that many organizations had to cope with at the beginning of the pandemic.

As expected, most of the participants teleworked, although only a small percentage had had previous experience with this work format. Our figures are slightly higher than those provided by Ahrendt et al. (2020), which indicated that in the June–July 2020 period, 52% of the Spanish population was teleworking. This abrupt shift to telework required an effort from both the workers and the organizations. The workers, most of whom had no previous experience with teleworking, proactively put their private context, own resources, and skills to respond to the new and unknown job demands. The organizations had to establish, urgently and reactively, work management measures.

Regarding demographic and organizational data (RQ1), the highest percentages of teleworkers were found among managers, employees aged over 46, employees with more than 10 years’ experience in the same company, and men. These results partially coincide in terms of age, given that already in 2019 the highest percentage of teleworkers appeared in the over-55 age group (INE, 2020). We can assume that most managers are in this age group, that in turn they will hold higher positions in their companies, and that they tend to be men—only 34% of managers in Spain are women and only 23% of women managers are CEOs (Grant Thorntorn, 2021). The characteristic profile of the teleworker described in our study corresponds to a middle-aged man with managerial responsibility. This profile usually has the necessary resources to carry out their task remotely, although they may not have the necessary digital skills. For this reason, organizations had to worry, not only about providing the necessary software for work, but also about equipping them with digital skills to manage work teams effectively.

In addition, organizations should pay special attention to women, given that, as pointed out by Grubanov-Boskovic et al. (2022) “as the COVID-19 pandemic unfolded, its adverse implications for gender equality started penetrating not only the dimension of paid work, but also the area of unpaid (domestic and childcare) work” (Grubanov-Boskovic et al., 2022, p. 2). It should be added how women have experienced this period in a particularly negative way, in relation to the imbalance produced by their work and personal obligations, and the effects on their mental health (Esteban-Gonzalo et al., 2020; Romeo et al., 2021).

The sector with the highest percentage of teleworking employees was education, public administration, and health, in companies with fewer than 250 employees, a finding that coincides with data on the percentage of workers who worked from home in 2019, prior to the pandemic (Hernández de Cos, 2020). It is important to consider that the sectors of education and public administration were those with the better conditions to deal with the situation of teleworking, even though their employees needed to use their own technological resources. Contrarily, healthcare sector was the one with the highest levels of frontline employees, but in addition, they had a high percentage of virtual work, allowed by different technological tools as virtual care technologies or wearable devices (Hurley and Popescu, 2021; Maxwell and Grupac, 2021; Walters and Kalinova, 2021).

To answer RQ2, the employees’ assessment of their remote working conditions was analyzed. Overall, the telework experience was valued as positive, with the best-considered variables being access to information needed for work, their colleagues’ attitude and efficiency, and work productivity, quality, and effectiveness. The assessment of access to information is confronted with the availability of technological means, which on many occasions were not provided by the companies, at least initially. In addition, it is relevant to note that the limits between work time and personal time were broken during the pandemic (Romeo et al., 2021). Therefore, the participants indicated their own time management as one of the worst valued aspects. Finally, their perception in relation to the positive results obtained may be due to a process of cognitive dissonance reduction (Festinger, 1962) derived from the effort made to achieve them.

When comparing results by sociodemographic and organizational variables, the greatest differences in the assessment of teleworking conditions was found by sectors. The employees who teleworked in the distribution and consumption sector, characterized by being the sector with the lowest percentage of teleworkers in our sample, were the ones who best valued the working conditions in which they teleworked. Despite this, their overall satisfaction was somewhat lower than that of employees in other sectors, except for education, public administration, and health. The latter sectors contained the highest percentage of teleworking employees, and in turn, those who worst valued the telework situation and their general satisfaction with this format. In addition, they agreed with employees in the industrial sector in assessing more negatively their colleagues’ attitude and efficiency while teleworking.

The results obtained in the fields of education, public administration, and health partially contradict previous studies carried out in Spain, where one of the main complaints voiced by teachers, both at basic educational levels (Trujillo Sáez et al., 2020) and in higher education (Romeo et al., 2021; Tasso et al., 2021), was the lack of technical resources, information, and institutional support. Contrarily, other studies have pointed out that teachers perceived benefits on the use of new technologies and instruments for education, even though they stated that the educational system was not prepared for that sudden change (Obrad and Circa, 2021). Nevertheless, in the case of Dutch public servants, our results are in the line of the results of De Vries et al. (2016). The authors evidenced negative effects from teleworking, including greater professional isolation and less organizational commitment on the days that they worked entirely from home.

Thirdly, the participants’ perception of the measures taken by their companies to cope with the pandemic was analyzed (RQ3). In general, the ratings were medium–high. All the measures were slightly better valued by teleworkers than non-teleworkers, with the exception of communication with clients, which was valued more positively by the latter. Health and prevention measures were another aspect given consideration. They were best valued among teleworkers, while they were the worst valued by non-teleworkers. This result ties in with the findings of the study of Rozentale et al. (2021) who pointed these organizational measures are one of the most important motivation tools by employees as the different sectors in Latvia, during the lockdown, and with the research carried out by the International Labor Organization [International Labor Organization (ILO), 2020] according to which:

Frontline workers, such as health care and emergency workers, but also those involved in the production of essential goods, in delivery and transportation, or in ensuring the security and safety of the population are facing many stressful situations at work as a result of the COVID-19 pandemic. Increased workloads, longer working hours, and reduced rest periods are a concern for most of them. In addition, they may be worried about getting infected at work and passing the virus to family, friends, and others at work, in particular if appropriate protective measures are not in place (p. 6).

In relation to how different groups coped with the new situation within the organization (managers, middle managers, and colleagues), they were all once again best valued by the teleworkers (RQ4). This result is related to our results in relation to the employees’ assessment about their colleagues’ attitude and efficiency. Again, it could be indicative of a mechanism to reduce cognitive dissonance, and it is related to the importance attributed to social support, which acts as a buffer against the situation of isolation caused by the pandemic. However, both teleworkers and non-teleworkers more highly rated their peers than their managers and middle managers. In this sense, a decrease in satisfaction is observed with increasing hierarchical distance. In addition, the perceptions of the employees who did not telework about their managers were worse than those of the employees who were teleworking. As almost all the managers teleworked, this result would be explained by a lack of equity as a result of social comparison processes (Festinger, 1954). The employees would attribute that the conditions of their managers were highly advantageous in terms of health and safety, both for them and for their families.

This result is linked to the decision trees developed to explain motivation (RQ5). Here, for both teleworkers and non-teleworkers, the variable that explained motivation was the ability of managers to cope with the situation. In this sense, our results are in line with Walker (2021) who evidenced more significant relationships between employee motivation and managers who provided helpful management and work organizational measures during COVID-19. Furthermore, in the case of teleworkers, the variable related to the results obtained doing this type of work is included.

Additionally, according to the ILO report (2020), these findings can be explained by the fact that workers may have been reluctant to ask for support or raise occupational safety and health concerns, or they may have adopted unhealthy working practices with the aim of pleasing managers and supervisors (for example, long working hours, increased workload). They may have also avoided taking time off work if they were sick, with the ensuing risk of infecting coworkers. This problem was more serious for those workers on short-term contracts or who had been hired under freelancing arrangements [International Labor Organization (ILO), 2020].

Finally, the employees who did not telework claimed they felt more highly motivated, even though their evaluation of the measures taken by the organization and their colleagues and superiors was more negative than that of teleworkers. However, it is worth bearing in mind that, although the difference is statistically significant, it only comes to one-tenth. Our results can be explained by the findings of Sarfraz et al. (2021). These authors found that even though professional isolation was an antecedent of lower levels of motivation among teleworkers, this relationship was moderated by access to communication-enhancing technology. In our sample, the lowest rated aspect of teleworking conditions was the availability of technological resources for working from home. In this sense, when employees felt that they did not have suitable technology to work from home, their levels of motivation decreased in comparison to non-teleworkers.

Hitka et al. (2021) found decreasing levels of motivation when comparing data before and during the pandemic in micro and small enterprises, with the decline being the result of perceptions of inequality, job insecurity, and difficulties in internal communication processes. In this research, non-teleworkers’ perceptions of these variables were worse than teleworkers’, but contrary to what might be expected, the former rated communication with clients (external communication) higher than teleworkers did.

To sum up, this research confirms that the situation resulting from the pandemic has forced many employees to telework, especially managers. Despite having no previous experience of working from home, they valued very positively both the measures adopted by their organizations and how other workers and managers dealt with the situation. It is important to bear in mind that, in part, these results may be due to an attempt to reduce the cognitive dissonance caused by the overexertion they had to make to cope with the situation. Contrary to expectations, employees who teleworked, despite giving these variables better ratings, displayed lower levels of motivation than those who did not telework. And as can be seen in the decision trees, their motivation was much more heavily conditioned by their perceptions of the productivity, quality, and effectiveness of their work.

The large amount of data obtained from this research offers a picture of the situation experienced by teleworkers and non-teleworkers during the pandemic in spring 2020. This data brings to light the importance of the role of managers in sustaining the motivation of their subordinates in times of crisis. Furthermore, our results permit the characterization of those employees with the highest levels of motivation in comparison with employees with medium or low levels. This has practical implications that could be useful for managers. In this sense, managers must be provided with the tools needed to cope with these situations and given communication strategies that enable them to keep in touch with those in their charge, thereby maintaining an atmosphere of trust and support. Likewise, employees must be informed of and able to value the results they obtain during emergency situations, especially those working from home, who, on occasions, may lose sight of the value of their work in detriment to their sense of importance and responsibility.

### Limitations and Future Research

Despite its contributions, this study is not without limitations. First of all, a single-item was used to measure the different aspects evaluated. Nevertheless, the research carried out by Ang and Eisend (2018), which examined 189 studies from eight previous meta-analyses, showed that when single-item measures are used, the results are the same as in those that use multiple items. Similarly, the study done by Bergkvist and Rossiter (2007) was one of the first to show empirically that one construct could predict another through a single or multiple items, and Rossiter (2002, 2011) developed the theory that it is not necessary to measure all constructs with multiple items, even for academic research. Additionally, Bean and Roszkowski (1995), Dillman et al. (1993), and Roszkowski and Bean (1990) found that the fewer questions a questionnaire had, the more likely participants were to respond willingly, with briefer questionnaires precluding boredom and fatigue among the respondents, and therefore improving the response rate.

Secondly, participants responded voluntarily in this research, and for that reason it could exist a selection bias that could explain the higher scores obtained, especially in motivation. Additionally, to avoid the identification of individual subjects, not all demographic data was collected in some organizations. This is the reason for the large amount of missing data, specially related to gender, age, and tenure, and the consequence may well have been that it was not sufficiently present in the classification trees to of significance when explaining motivation. Besides, sector categories were too broad and included in the same group sector that could have differential functioning, especially in the case of education, public administration, and health, which have been included into the same category. Nevertheless, the valid data was relevant enough to obtain significant results.

Thirdly, the instrument used was a self-administered questionnaire, which can facilitate common method variance (Podsakoff et al., 2003). The exploratory research design was aimed at obtaining massive data at a complicated time for the participating organizations, and for teleworkers and other employees. For this reason, the instrument had to be constructed in a way that facilitated responses but at the same time offered organizational decision-makers relevant information and keys for intervention. In this sense, to describe the measures implemented by the organization, broad terms referring to management systems were used. This fact did not allow to specify the actions on which the employee focuses when assess them. The use of mixed methods should be considered for future research in order to carry out a deeper assessment of the conditions affecting motivation in times of crisis.

Fourthly, we used a sectional design. For that reason, our results, obtained by means of decision trees, cannot explain causality. Future research should include at least two measures to have a deeper insight into causality evidence.

Fifthly, in relation with the classification trees, our results were good predictors of having low or medium levels of motivation, but not so good for the highest levels. In this sense, it is necessary to consider other variables to explain better how employees reach higher levels of motivation in times of COVID. Nevertheless, these results have practical implications, as they give managers keys for the improvement of motivation.

Finally, it should be taken into account that the data collected refer to the state of emergency at the beginning of the pandemic in spring 2020 and that it cannot be generalized to other later times or situations. Future research is needed to explore the conditions that influence the motivation of teleworkers and non-teleworkers, in sectors different from those analyzed in this research, and with a more specific segmentation of all the sectors.

## Data Availability Statement

The raw data supporting the conclusions of this article will be made available by the authors, without undue reservation.

## Ethics Statement

Ethical review and approval was not required for this current study on human participants in accordance with the local legislation and institutional requirements. Written informed consent for participation was not required for this study in accordance with the national legislation and the institutional requirements. The study which produced the data on which this study is based was reviewed and approved by E-Motiva Consulting. The patients/participants provided their written informed consent to participate in this study.

## Author Contributions

MR and MY-B contributed to theory development, research design, data analyses, and the discussion. LB contributed to the data analysis. All authors contributed to the article and approved the submitted version.

## Conflict of Interest

The authors declare that the research was conducted in the absence of any commercial or financial relationships that could be construed as a potential conflict of interest.

## Publisher’s Note

All claims expressed in this article are solely those of the authors and do not necessarily represent those of their affiliated organizations, or those of the publisher, the editors and the reviewers. Any product that may be evaluated in this article, or claim that may be made by its manufacturer, is not guaranteed or endorsed by the publisher.
